# Reduced Proprotein convertase subtilisin/kexin 9 (PCSK9) function increases lipoteichoic acid clearance and improves outcomes in Gram positive septic shock patients

**DOI:** 10.1038/s41598-019-46745-0

**Published:** 2019-07-22

**Authors:** Alex K. K. Leung, Kelly Roveran Genga, Elena Topchiy, Mihai Cirstea, Tadanaga Shimada, Chris Fjell, James A. Russell, John H. Boyd, Keith R. Walley

**Affiliations:** Centre for Heart Lung Innovation, St. Paul’s Hospital, The University of British Columbia, Vancouver, BC Canada

**Keywords:** Toll-like receptors, Sepsis, Bacterial infection

## Abstract

Previous studies have shown lipopolysaccharide from Gram-negative bacteria is cleared from the circulation via LDL receptors on hepatocytes, which are downregulated by PCSK9. Whether clearance of Gram positive bacterial lipoteichoic acid (LTA) shows similar dependence on PCSK9, and whether this is clinically relevant in Gram positive human sepsis, is unknown. We examined survival data from three cohorts of patients who had Gram positive septic shock (n = 170, n = 130, and n = 59) and found that patients who carried a *PCSK9* loss-of-function (LOF) allele had significantly higher 28-day survival (73.8%) than those with no LOF alleles (52.8%) (p = 0.000038). Plasma clearance of LTA was also found to be increased in PCSK9 knockout mice compared to wildtype control mice (p = 0.002). In addition, hepatocytes pre-treated with recombinant wildtype PCSK9 showed a dose-dependent decrease in uptake of fluorescently-labeled LTA (p < 0.01). In comparison to wildtype PCSK9, hepatocytes pre-treated with 3 different LOF variants of recombinant PCSK9 showed an increase in LTA uptake. This study shows the clearance of LTA follows a similar route as lipopolysaccharide, which is dependent on hepatic LDL receptors. This has important implications in health as strategies aimed at inhibiting PCSK9 function may be an effective treatment option for both Gram-positive and negative sepsis.

## Introduction

Lipopolysaccharide (LPS) from the outer membrane of Gram negative bacteria is a potent activator of Toll-like receptor 4 and triggers a septic inflammatory response^1–3^. LPS is incorporated within lipoprotein particles including HDL, LDL, and VLDL and is then cleared from plasma via LDL receptors expressed on hepatocytes^4–6^. Proprotein convertase subtilisin/kexin 9 (PCSK9) binds the LDL receptor, targets it for degradation, and therefore decreases cell surface expression of LDL receptors on hepatocytes. Thus, increased PCSK9 levels observed during sepsis^7^ results in fewer LDL receptors and, consequently, decreased LPS clearance^7,8^. Conversely, decreased PCSK9 function results in increased LPS clearance, a decreased septic inflammatory response and improved septic shock survival^7,8^.

Gram positive bacteria lack LPS. Lipoteichoic acid (LTA) is often viewed as an analog from Gram positive bacteria as it similarly triggers the NF-κB signalling pathway through activation of Toll-like Receptors (TLR2). Like LPS, LTA is also incorporated into lipoprotein particles including HDL, LDL, and VLDL^9^. In addition, the uptake of LTA from Gram positive organisms into hepatocytes is similarly regulated by PCSK9 and is mediated through the LDL receptor pathway^10^. Whether PCSK9 could alter outcome in Gram positive human sepsis via this pathway is unknown. This is a clinically relevant issue because, in most patients who present with bacterial sepsis, characterization of the specific bacterial pathogen takes hours to days while initiation of therapy to kill and clear bacteria must be initiated immediately for optimal patient outcomes. Rapid clearance of bacterial lipids is important following the surge of these toxic lipids as a result of antibiotic-induced lysis of the bacterial membrane and release of its components into the circulation^11^.

In view of the similarities between LTA and LPS molecular structure and their similar transport via lipoproteins, we postulated that PCSK9 affects the outcome of Gram positive sepsis in humans by regulating the clearance of LTA through the hepatic LDL receptor pathway. We first addressed this hypothesis by determining whether *PCSK9* LOF genotype was associated with improved outcomes in patients who had Gram positive septic shock and, further, whether this benefit was eliminated in the subset of patients who also carried an *LDL receptor* genetic variant that would render the LDL receptor insensitive to PCSK9 regulation. We then sought to determine whether clearance of LTA via the LDL receptor pathway^10^ could account for this finding.

## Results

### Clinical outcomes in septic shock cohorts

**Figure 1:**
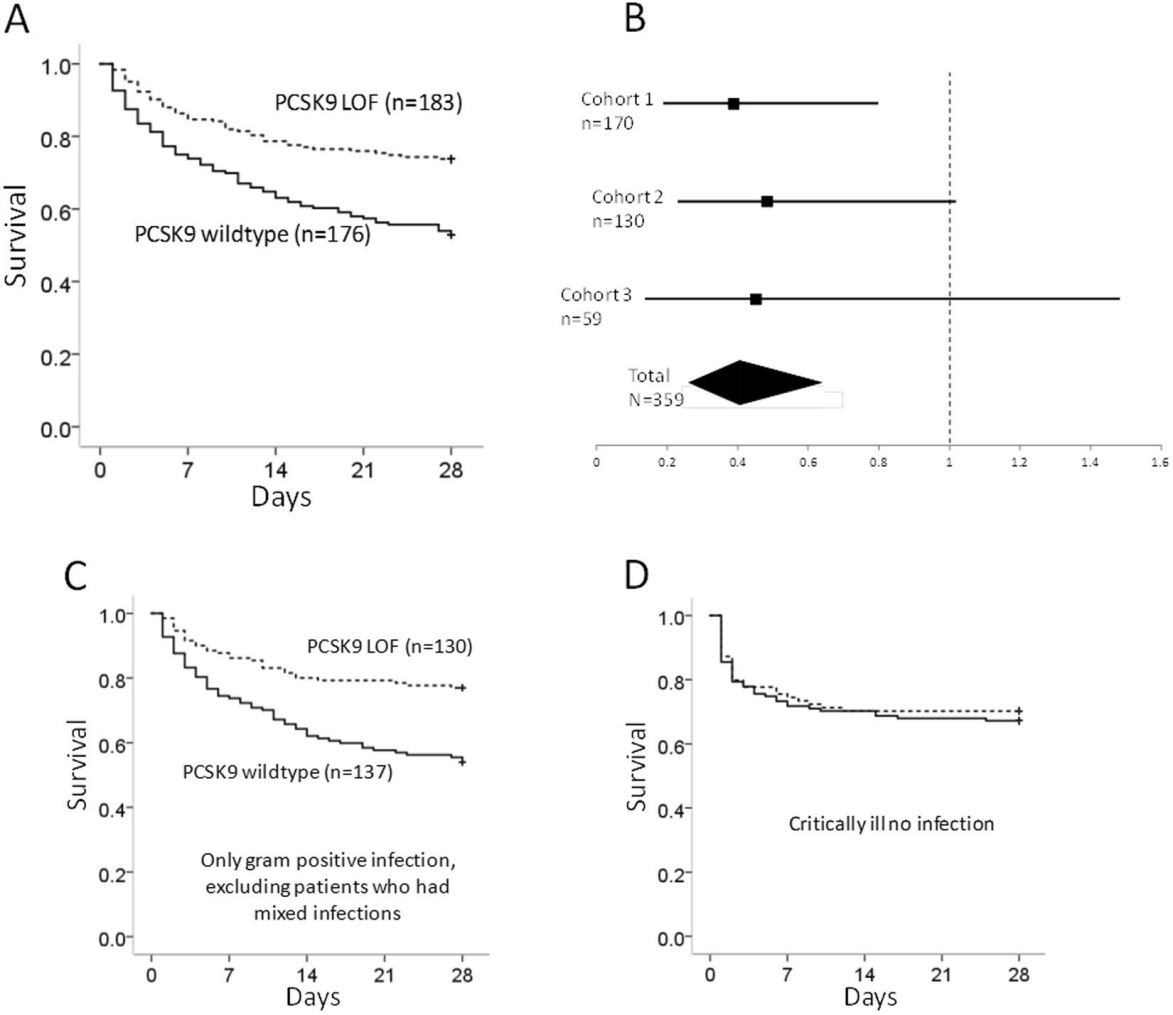
(**A**) Survival curves for septic shock patients with Gram positive bacterial infection. Patients who carried at least one Loss-Of-Function (LOF) allele of *PCSK9* (dashed line) had increased 28-day survival compared to patients with the wildtype *PCSK9* genotype (solid line) (p = 0.000038). (**B**) Odds ratios and 95% confidence intervals for LOF effect on 28-day mortality, using logistic regression, are shown for all three septic shock cohorts and the combined total cohort (black diamond shape). Each cohort had a similar point estimate of LOF effect on 28-day mortality. Patients who carried at least one LOF allele of PCSK9 (dashed line) had decreased 28-day mortality compared to patients with the wildtype PCSK9 genotype (p < 0.0001). (**C**) Survival curves for patients who only had confirmed Gram positive infection, excluding those who had mixed Gram positive and other pathogen infections (p < 0.0001 by log rank test). (**D**) Survival curves for non-septic critically ill patients show no difference in 28-day survival by PCSK9 genotype.

PCSK9 reduces the clearance of LPS from the circulation via the LDL receptor^7^ and patients with *PCSK9* LOF variants have improved outcomes in sepsis^8^. Gram positive bacteria, however, do not contain LPS. To determine whether *PCSK9* LOF genotypes would confer improved survival in patients with Gram positive sepsis, we examined patients with culture positive Gram positive sepsis from three independent septic shock cohorts; cohort 1 n = 170^12^, cohort 2 n = 130^13^, and cohort 3 n = 59. Baseline characteristics are shown in Table 1. Patients who carried a *PCSK9* LOF allele had significantly higher 28-day survival (73.8%) than those with no LOF alleles (52.8% survival, p = 0.000038) (Fig. 1A). All three cohorts were combined in a secondary logistic regression analysis adjusted for age, sex, medical versus surgical diagnosis, and ancestry (Caucasian or not) (Fig. 1B). *PCSK9* LOF was associated with significantly increased 28-day survival (Odds ratio of mortality for *PCSK9* LOF 0.406, 95% CI 0.257–0.639, p < 0.0001). Survival decreased with age (p = 0.003), but did not differ by sex (p = 0.99), by medical versus surgical diagnosis (p = 0.143), or by ancestry (p = 0.139).

**Table 1:** Baseline characteristics of patients with Gram positive sepsis by PCSK9 genotype.

	No LOF	PCSK9 LOF genotype	p value
**All Gram positive sepsis (including mixed infections)**			
	n = 176	n = 183	
Age	59.4 ± 16.6	57.1 ± 16.0	0.18
% Female	36%	42%	0.32
APACHE II Score	26.4 ± 7.9	25.8 ± 7.7	0.48
% with Surgical Diagnosis	23%	17%	0.17
**Only Gram positive sepsis (excluding mixed infections)**			
	n = 137	n = 130	
Age	59.3 ± 17.2	56.0 ± 16.2	0.11
% Female	36%	38%	0.94
APACHE II Score	25.8 ± 7.6	25.8 ± 7.3	0.98
% with Surgical Diagnosis	23%	14%	0.067

Mean ± Standard Deviation.

Loss Of Function (LOF).

We repeated this analysis for patients with only Gram positive infection, excluding patients who had mixed infections (baseline characteristics Table 1, bottom panel). Patients who carried a *PCSK9* LOF allele (n = 130) had significantly greater survival over 28 days (76.9%) compared to patients who did not carry a LOF allele (n = 137) with a 28 day survival of 54.0% (p < 0.0001) (Fig. 1C). In the adjusted logistic regression analysis *PCSK9* LOF remained associated with increased 28 day survival (odds ratio of mortality 0.326, 95% CI 0.181–0.586, p = 0.00018). A further secondary analysis was restricted to those patients who met the Sepsis-3 definition of septic shock (lactate >2, n = 154). *PCSK9* LOF was again associated with increased 28-day survival (p = 0.000072). *PCSK9* LOF remained significantly associated with increased survival for different Gram positive infection including lung sources (p = 0.004, n = 189) and extrapulmonary sources (p = 0.003, n = 170).

For culture-negative septic shock (no Gram positives, no Gram negatives, no fungi) there was no statistically significant difference in 28-day survival by *PCSK9* genotype (survival *PCSK9* wildtype 63.7%, n = 388, PCSK9 LOF 68.9%, n = 325, p = 0.13). In addition, cohort 2 included critically ill patients who were not septic. These patients were genotyped for a *PCSK9* haplotype tag SNP rs644000; the minor allele tags *PCSK9* haplotypes containing LOF SNPs (Online supplement). *PCSK9* LOF was not associated with different outcome in these patients (Fig. 1D).

In addition to improved overall survival, Gram positive septic shock patients who carried a *PCSK9* LOF allele had a statistically significantly greater median days alive and free of cardiovascular (p = 0.001), respiratory (p = 0.025), renal (p < 0.001), hematologic (p = 0.004), hepatic (p = 0.002) and neurologic dysfunction (p = 0.005) (Table 2). Patients with a *PCSK9* LOF allele also had more days alive and free of vasopressors (p = 0.003), mechanical ventilation (p = 0.016) and renal replacement therapy (p < 0.001) (Table 2).

**Table 2:** Days Alive and Free of organ dysfunction and artificial organ support by Loss-Of-Function (LOF) genotype of *PCSK9*. More days alive and free of organ failure or organ support is a beneficial outcome so *PCSK9* LOF is associated with less organ failure.

*PCSK9 LOF*	No LOF	LOF	*P*
	(n = 154)	(n = 146)	
**Days alive and free**			
Organ dysfunction			
Cardiovascular	9 (0–22)	20 (3–25)	0.001
Respiratory	2 (0–16)	6 (0–20)	0.025
Renal	12 (1–28)	25 (10–28)	<0.001
Hematologic	21 (2–28)	26 (10–28)	0.004
Hepatic	19 (3–28)	28 (10–28)	0.002
Neurologic	12.5 (1–24)	19 (6–27)	0.005
Artificial support			
Vasopressor use	13 (0–23)	21 (3–25)	0.003
Ventilator	2 (0–18)	12 (0–21)	0.016
Renal replacement therapy	14 (2–28)	28 (12–28)	<0.001
Overall days alive	28 (1–28)	28 (1–28)	<0.0005

Data are median (interquartile range).

*p* values were calculated using a MannWhitney U test.

Organ dysfunction was recorded as positive if the patient met the SOFA organ dysfunction criteria (>1) that day with the platelet threshold of 80. The number of days out of 28 where a patient is alive with no documented organ dysfunction is a Day Alive and Free of organ dysfunction.

Organ failure scores were only available for cohort 1 and 2, hence the lower n compared to Table 1.

To test for evidence of involvement of the LDL receptor in survival and *PCSK9* genotype effects, as previously reported^8^, we repeated the primary survival analysis in patients from cohort 1 who had *LDL receptor* rs688 genotyped and were homozygous for the minor allele, a variant that renders the LDL receptor unresponsive to PCSK9. Survival curves for patients with and without *PCSK9* LOF were now indistinguishable (p = 0.96, Supplemental Fig. 1) and all the benefit of *PCSK9* LOF was confined to patients who carried at least one functional allele of rs688 (p = 0.023). These data are consistent with involvement of the LDL receptor in the *PCSK9* effect.

### Plasma Clearance of LTA

To test whether PCSK9 affects the systemic clearance of LTA, we compared the plasma concentration of LTA in *Pcsk9* knockout (*Pcsk9*^−/−^) and their wildtype litter-mates following an intravenous administration of BODIPY-FL-LTA. Six hours after the administration of BODIPY-FL-LTA, *Pcsk9*^−/−^- mice had 33% lower plasma BODIPY-FL-LTA fluorescence as compared to wildtype litter-mates (3.1 × 10^7^ versus 2.0 × 10^7^, p = 0.002), indicating increased plasma clearance of LTA in *Pcsk9*^−/−^ mice (Supplemental Fig. 2). This suggests that decreased PCSK9 function enhances plasma clearance of LTA through an increase in hepatocellular uptake.

### Uptake of LTA by hepatocytes

**Figure 2:**
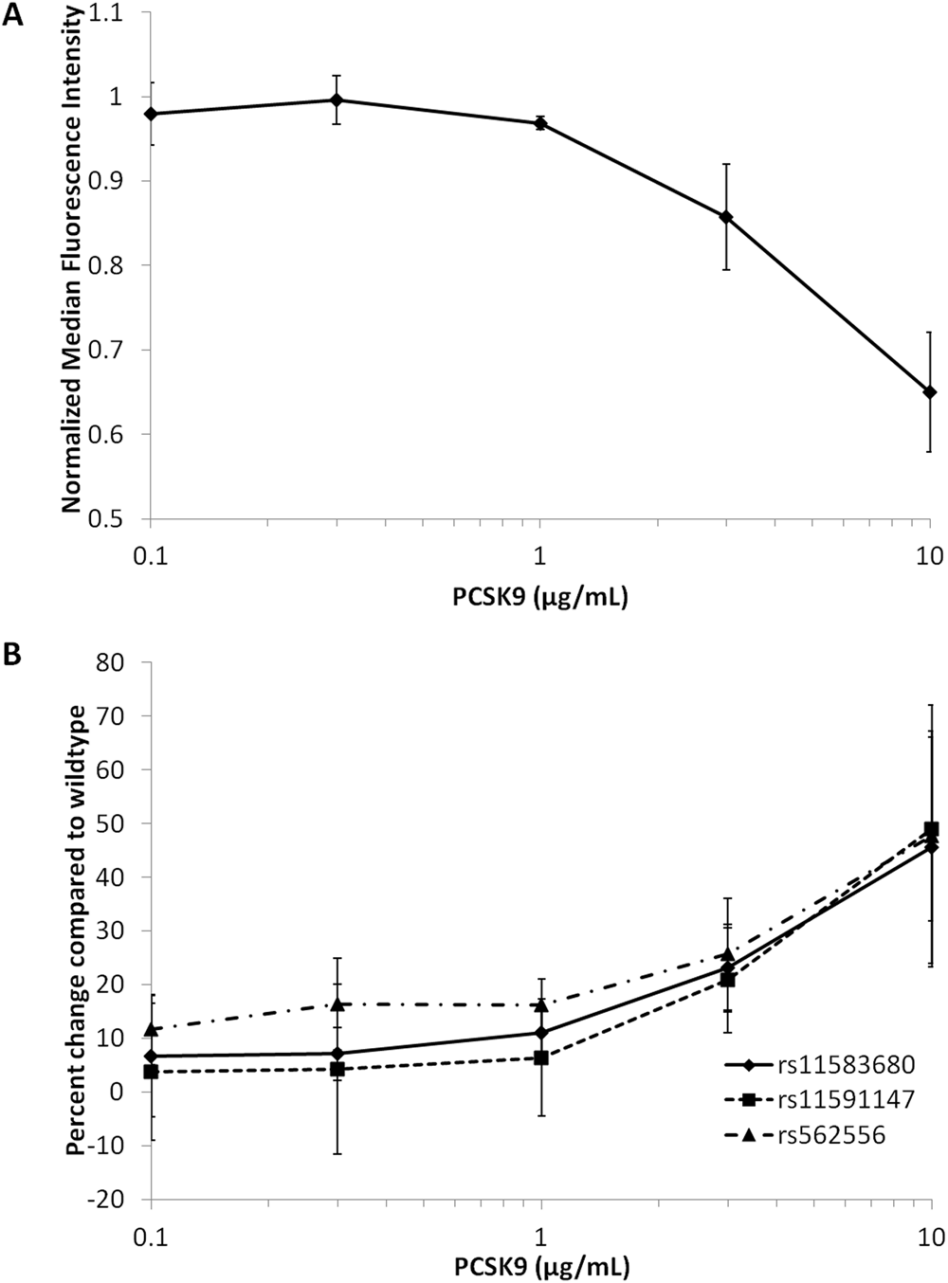
PCSK9 function is negatively correlated with the uptake of lipoteichoic acid by hepatocytes. (**A**) HepG2 hepatocytes were pre-treated with various concentrations of recombinant wildtype PCSK9 two hours before the treatment of the fluorescently labeled LTA (BODIPY-FL-LTA, 1 μg/mL). After 24 hours of LTA treatment, cells were analyzed by flow cytometry. Mean fluorescence intensity were normalized to cells without PCSK9 treatment. (**B**) Same as experiment as in (**A**) but HepG2 hepatocytes were treated with 3 different PCSK9 LOF variants, rs11583680, rs11591147 and rs562556. Results are shown as percent change in LTA uptake as compared cells treated with wildtype PCSK9 at the same concentration. Error bars are Standard Deviations; n = 3 experiments per data point.

Previous studies have demonstrated the ability of LTA to bind to lipoproteins^9^, we therefore compared the cellular uptake of fluorescently labeled LTA in the presence of PCSK9. PCSK9 dose-dependently decreased the uptake of LTA by HepG2 hepatocytes with over 30% reduction in uptake at 10 μg/mL of PCSK9 (Fig. 2A) (p < 0.01). In contrast, cells treated with the *PCSK9* LOF variants rs11583680 (A53V) and rs11591147 (R46L), and rs562556 (V474I) showed an increase in the uptake of the fluorescently labeled LTA when compared to the wildtype (Fig. 2B) (p < 0.01 for all *PCKS9* LOF variants).

**Figure 3:**
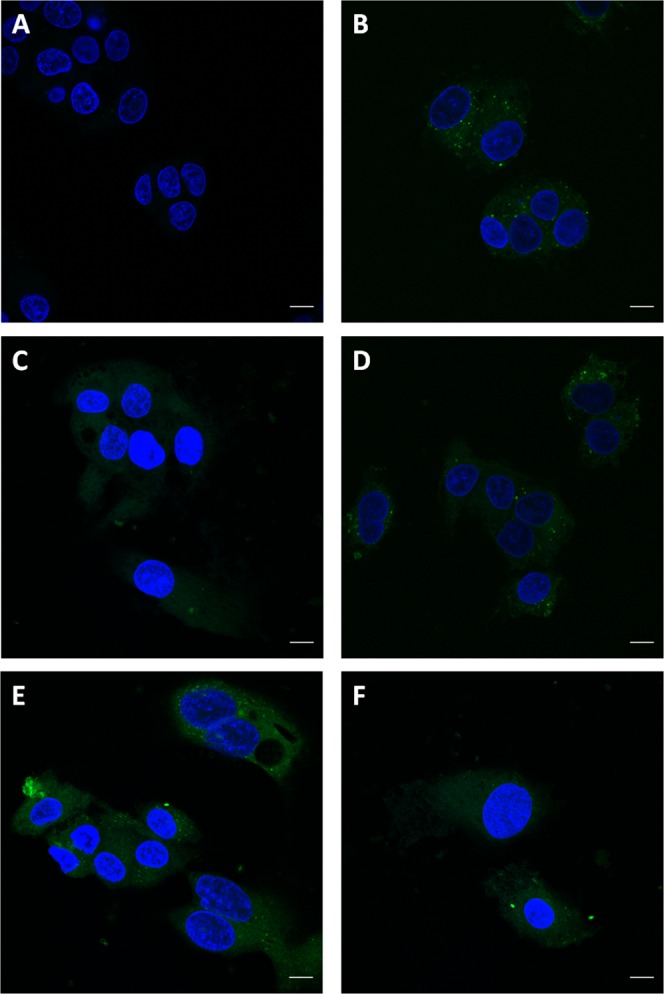
Influence of PCSK9 on the cellular uptake of lipoteichoic acid. HepG2 hepatocytes were pre-treated with 10 μg/mL of either recombinant wildtype PCSK9 or 3 different PCSK9 LOF variants, rs11583680, rs11591147 and rs562556, 4 hours before the treatment of the fluorescently labeled LTA (BODIPY-LTA, 2 μg/mL). (**A**) Cells untreated with BODIPY-FL-LTA were imaged as a negative control. Cells treated with BODIPY-FL-LTA along (**B**) without PCSK9 or with (**C**) wildtype, (**D**) rs11583680, (**E**) rs11591147 and (**F**) rs562556 PCSK9. Nuclei were stained with Hoescht 33342 shown in blue and BODIPY-FL-LTA fluorescence is shown in green. Scale bars represent 10 µm.

We examined cellular uptake of the BODIPY-FL-LTA using confocal microscopy. Cells were pre-treated with 10 μg/mL of either recombinant wildtype PCSK9 or LOF variants of PCSK9 followed by a continuous treatment with BODIPY-FL-LTA for 4 hours. Confocal images correlated with the flow cytomety data as cells treated with the PCSK9 LOF variants clearly showed higher cellular uptake than wildtype PCSK9 (Fig. 3). In addition, the punctate staining pattern of LTA suggests the LTA were localized within endocytic compartments. Thus increased hepatocellular uptake of LTA occurs in the presence of the PCSK9 LOF variants as compared to the wildtype.

## Discussion

We found that patients with one or more *PCSK9* LOF alleles have better outcomes from Gram positive sepsis which may be explained, in part, by our observation that hepatocytes treated with LOF variants of recombinant PCSK9 have increased uptake of LTA *in vitro* compared to cells treated with wild-type PCSK9. Our results support, but do not prove, the hypothesis that LTA is cleared from the circulation through the hepatic LDL receptor pathway which is negatively regulated by PCSK9, similar to the clearance of LPS from Gram negative bacteria.

Gram positive organisms, which lack LPS in their membrane, are responsible for as many as half of the incidents of sepsis in intensive care units^14–16^. LTA from Gram positive bacteria triggers the innate immune response through activation of Toll-like receptors, including TLR2^17–21^. LTA is structurally similar to LPS from Gram negative bacteria and both pathogen lipids have been shown to bind to various lipoproteins in the circulation^5,6,9,22^. Binding of LTA to lipoproteins has been shown to reduce its ability to activate macrophages^23^. However, it is unknown whether LTA from Gram positive bacteria follows the same clearance pathway as LPS.

We found that LTA uptake by hepatocytes is dose-dependently reduced by PCSK9. PCSK9 LOF variants result in greater LTA uptake by hepatocytes, compared to wildtype PCSK9 at the same concentrations. The same PCSK9 LOF variants in human sepsis are associated with increased survival and decreased organ dysfunction. Supporting this *in vitro* observation, *Pcsk9*^−/−^ mice also showed enhanced plasma clearance of intravenously administered LTA. Finally, in patients who carry an LDL receptor genetic variant that is unresponsive to PCSK9, there is no observed benefit of *PCSK9* LOF. These results support the hypothesis that LTA is cleared from circulation through the hepatic LDL receptor pathway which is negatively regulated by PCSK9.

This study, along with previous work^8^, suggests that decreased PCSK9 function is associated with improved clinical outcomes, possibly by promoting the hepatic clearance of both LTA and LPS through the upregulation of hepatic LDL receptors. Thus, if PCSK9 inhibition had a similar beneficial effect on clinical outcomes as PCSK9 LOF genotype, it may not be necessary to identify the causative organism as Gram positive or Gram negative when considering PCSK9 inhibition as a novel early intervention for sepsis. Both Gram positive and Gram negative sepsis have similar clinical presentations and the identification of the causative organism often requires 24 to 48 hours.

Numerous approaches to inhibit PCSK9 have been developed for the treatment of hypercholesterolemia, including anti-PCSK9 monoclonal antibodies^24,25^, small peptide inhibitors of PCSK9^26,27^, and small interfering RNA^28,29^. Monoclonal antibodies against PCSK9 which were developed for the treatment of familial hypercholesterolemia have been shown to decrease free PCSK9 levels in the plasma almost immediately along with a significant drop in plasma LDL-cholesterol levels as early as 1 day after administration^25^. They work by allosterically blocking the site of interaction between PCSK9 and LDL receptor. One or more of the above approaches could prove beneficial in future trials in sepsis due to Gram negative^8^ or Gram positive infection.

Our study has a number of limitations. Patients with Gram positive sepsis also have circulating endotoxin and it has been demonstrated that high levels of endotoxins in the circulation can be detected in patients without Gram-negative bacteremia^30,31^. The permeability of intestinal tight junctions is decreased in many types of sepsis, which may cause additional increases of endotoxin into the blood stream^32,33^. In addition, antibiotics can accelerate the release of endotoxin into circulation^34,35^. Thus, it is possible that some of the observed benefit may be related to increased clearance of Gram-negative endotoxin; not all of the benefit relates to increased clearance of LTA. We addressed this concern by genotyping culture negative septic patients who similarly have increased circulating endotoxin levels. *PCSK9* LOF had no effect in these patients supporting the conclusion that increased clearance of LTA plays a significant role in explaining improved clinical outcome in patients with *PCSK9* LOF genotypes. Alternatively, some culture negative infections may have been viral and therefore did not have the same burden of pathogen lipids to be cleared via a PCSK9-sensitive pathway. In addition to LTA, the cell wall of the Gram positive bacteria contains other potent TLR2 agonists, including peptidoglycan and other bacterial lipoproteins^15^. It would be interesting to extend the current study to include these Gram positive bacterial cell wall components to examine whether their clearance is similarly dependent on the LDL receptor and PCSK9 and therefore contributing to the improved survival observed in *PCSK9* LOF patients. Other potential explanations for our results include the possibility that PCSK9 LOF has other effects. For example, in cecal ligation and puncture models of murine sepsis both *Pcsk9* knockout and treatment of mice with PCSK9 antibody reduced measures of bacterial load^8,36^. Thus, PCSK9 may have additional effects related to bacterial killing. There are also evidence PCSK9 may enhance the infectivity of Hepatitis C Virus^37^ and increase the pro-inflammatory response of macrophages^38^. Therefore, it cannot be excluded that reduced circulating PCSK9 levels may have a favourable impact on bacterial sepsis outcomes, independent from pathogen lipid clearance^39^. Our results were limited to septic shock patients, possibly because the burden of pathogen lipids was higher than in less severe sepsis.

In summary we found that PCSK9 LOF was associated with increased survival and decreased organ dysfunction in patients with Gram positive septic shock. The clearance of LTA from the systemic circulation appears to follow a similar route as LPS and the uptake of Gram positive bacterial LTA by hepatocytes is inhibited by PCSK9. This may be the explanation for the improved clinical outcomes that we observed in patients with *PCSK9* LOF variants. PCSK9 inhibition should be assessed in sepsis trials of patients who have Gram positive or Gram negative infection.

## Methods

### Study population

Patients who had septic shock due to Gram positive infection from the Vasopressin and Septic Shock Trial (VASST)^12^ recruited from 2001–2006 (cohort 1), the previously published St. Paul’s Hospital (SPH) cohort recruited from 2000–2004^13^ (cohort 2), and a third previously unstudied cohort recruited at SPH from 2004–2009 (cohort 3) were included in this study. Cohort 2 also included contemporaneously recruited non-septic critically ill patients who are used in this study as a control cohort. Inclusion criteria and human genotyping are described elsewhere and in the online supplement^12,40^. Gram positive infection was defined as Gram positive organisms growing within 5 days of inclusion from cultures which were judged by the physicians to be a clinically significant infection. We included patients who had Gram positive infection even if they also had positive cultures for Gram negative or fungal organisms.

We genotyped the most common *PCSK9* missense LOF variants (minor allele frequency, ≥0.5%), rs11591147 (R46L), rs11583680 (A53V) and rs562556 (G670E), the Gain-Of-Function SNP rs505151, and the *LDL Receptor* SNP rs688. Patients were categorized as LOF if they carried one or more minor alleles of any of the 3 *PCSK9* LOF SNPs and no copies of the minor allele of the Gain-Of-Function SNP.

### Ethics

The research ethics board of the University of British Columbia and St. Paul’s Hospital approved this study (H02-50076) and research ethics boards of all 27 participating institutions approved the VASST trial and written informed consent was obtained from all patients or their authorized representatives. All human studies were carried out in accordance with the approved guidelines. All animal studies were approved by the University of British Columbia animal ethics committee and conformed to the National Institutes of Health (NIH) *Guide for the Care and Use of Laboratory Animals*.

### Plasma clearance of LTA in animal models

*Pcsk9* knockout mice and wildtype litter-mate controls (n = 24, males and females, 20–30 weeks old,) were injected intravenously via the tail vein with BODIPY-LTA at 0.3 mg/kg. At 6 hours after LTA treatment, blood was collected by cardiac puncture and relative levels of BODIPY-FL-LTA in plasma were determined by measuring its fluorescence at λ_ex/em_ = 500/525 nm using the Spectramax i3 fluorescent plate reader.

### Flow cytometry

HepG2 hepatocytes were seeded into a 24-well plate and grown to 80% confluence in DMEM supplemented with 20% human plasma from pooled healthy donors. Cells were pre-treated with recombinant wild-type human PCSK9, or various loss-of-function PCSK9 variants for 2 h prior to treatment with BODIPY-labeled LTA. The cells were collected, washed and analysed via flow cytometry (GalliosTM Flow Cytometer, Beckman Coulter, Brea, CA) after 24 hrs of treatment. Cells were gated via forward and side scatter for viability using previously determined parameters. Autofluorescence of untreated cells was subtracted to determine the fluorescence level resulting from the uptake of the LTA conjugate. Data processing was performed using Kaluza Analysis 1.3 software (Beckman Coulter, Brea, CA).

### Confocal microscopy

HepG2 hepatocytes seeded onto coverslips were first pre-treated with 10 μg/mL of either wild-type or loss-of-function variants of human PCSK9 corresponding to the patient LOF genotypes (see above) for 4 hrs followed by treatments with BODIPY-labeled LTA for 4 hrs. The culture medium for the duration of the experiment was DMEM supplemented with 10% fetal bovine serum. Cells were fixed with 4% paraformaldehyde and examined with confocal microscopy (Zeiss LSM 880). Fluorochromes were excited at 405 nm (Hoescht 33342) and 488 nm (BODIPY-FL) and images were collected with a 62x oil-immersion objective lens.

### Statistical analysis

For human septic shock our primary analysis of 28-day survival used a log rank test. A secondary analysis, to adjust for the influence of confounding, used logistic regression to examine 28-day survival by *PCSK9* genotype, including the covariates of age, gender, medical versus surgical diagnosis, and Caucasian ancestry in the statistical model. A secondary analysis included only those patients with only Gram positive infection, excluding patients who had mixed infections. We tested for differences in days alive and free of organ dysfunction using a non-parametric Mann-Whitney U test. We tested for differences in LTA clearance in male and female mice using ANOVA stratified by sex. We used analysis of variance to test for differences PCSK9 effect over a dose range *in vitro* using ANOVA. We used SPSS (version 23, IBM) for all analyses and considered p < 0.05 to be statistically significant.

## Supplementary information

Supplemental Information

## Data Availability

The data in this study are available from the corresponding author on reasonable request.

## References

[1] Qureshi ST (1999). Endotoxin-tolerant mice have mutations in Toll-Like Receptor 4 (Tlr4). J. Exp. Med..

[2] Poltorak A (1998). Defective LPS signaling in C3H/HeJ and C57BL/10ScCr mice: mutations in Tlr4 gene. Science.

[3] Dunzendorfer S, Lee H-K, Soldau K, Tobias PS (2004). TLR4 is the signaling but not the lipopolysaccharide uptake receptor. J. Immunol..

[4] Topchiy E (2016). Lipopolysaccharide is cleared from the circulation by hepatocytes via the low density lipoprotein receptor. PLoS One.

[5] Levels JHM, Abraham PR, Van Den Ende A, Van Deventer SJH (2001). Distribution and kinetics of lipoprotein-bound endotoxin. Infect. Immun..

[6] Levels JHM (2005). Lipopolysaccharide is transferred from high-density to low-density lipoproteins by lipopolysaccharide-binding protein and phospholipid transfer protein. Infect. Immun..

[7] Boyd JH (2016). Increased plasma PCSK9 levels are associated with reduced endotoxin clearance and the development of acute organ failures during sepsis. J. Innate Immun..

[8] Walley KR (2014). PCSK9 is a critical regulator of the innate immune response and septic shock outcome. Sci. Transl. Med..

[9] Levels JHM, Abraham PR, Van Barreveld EP, Meijers JCM, Van Deventer SJH (2003). Distribution and kinetics of lipoprotein-bound lipoteichoic acid. Infect. Immun..

[10] Grin PM (2018). Low-density lipoprotein (LDL) - dependent uptake of Gram-positive lipoteichoic acid and Gram-negative lipopolysaccharide occurs through LDL receptor. Sci. Rep..

[11] Van Langevelde P (1998). Antibiotic-induced release of lipoteichoic acid and peptidoglycan from Staphylococcus aureus: Quantitative measurements and biological reactivities. Antimicrob. Agents Chemother..

[12] Russell JA (2008). Vasopressin versus norepinephrine infusion in patients with septic shock. N. Engl. J. Med..

[13] Nakada T (2010). β _2_ -Adrenergic Receptor Gene Polymorphism Is Associated with Mortality in Septic Shock. Am. J. Respir. Crit. Care Med..

[14] Kengatharan KM, De Kimpe S, Robson C, Foster SJ, Thiemermann C (1998). Mechanism of gram-positive shock: identification of peptidoglycan and lipoteichoic acid moieties essential in the induction of nitric oxide synthase, shock, and multiple organ failure. J. Exp. Med..

[15] Cohen J (2002). The immunopathogenesis of sepsis. Nature.

[16] Weber JR, Moreillon P, Tuomanen EI (2003). Innate sensors for Gram-positive bacteria. Curr. Opin. Immunol..

[17] Schröder NWJ (2003). Lipoteichoic acid (LTA) of Streptococcus pneumoniae and Staphylococcus aureus activates immune cells via Toll-like receptor (TLR)-2, lipopolysaccharide-binding protein (LBP), and CD14, whereas TLR-4 and MD-2 are not involved. J. Biol. Chem..

[18] Michelsen KS (2001). The role of toll-like receptors (TLRs) in bacteria-induced maturation of murine dendritic cells (DCs): Peptidoglycan and lipoteichoic acid are inducers of DC maturation and require TLR2. J. Biol. Chem..

[19] Schwandner R, Dziarski R, Wesche H, Rothe M, Kirschning CJ (1999). Peptidoglycan- and lipoteichoic acid-induced cell activation is mediated by Toll-like receptor 2. J. Biol. Chem..

[20] Morath S, Stadelmaier A, Geyer A, Schmidt RR, Hartung T (2002). Synthetic lipoteichoic acid from Staphylococcus aureus is a potent stimulus of cytokine release. J. Exp. Med..

[21] Ginsburg I (2002). Role of lipoteichoic acid in infection and inflammation. Lancet Infect. Dis..

[22] Levels JHM, Lemaire LCJM, van den Ende AE, van Deventer SJH, van Lanschot JJB (2003). Lipid composition and lipopolysaccharide binding capacity of lipoproteins in plasma and lymph of patients with systemic inflammatory response syndrome and multiple organ failure. Crit. Care Med..

[23] Grunfeld C (1999). Lipoproteins inhibit macrophage activation by lipoteichoic acid. J. Lipid Res..

[24] Dias CS (2012). Effects of AMG 145 on low-density lipoprotein cholesterol levels: Results from 2 randomized, double-blind, placebo-controlled, ascending-dose phase 1 studies in healthy volunteers and hypercholesterolemic subjects on statins. J. Am. Coll. Cardiol..

[25] Stein EA (2012). Effect of a monoclonal antibody to PCSK9 on LDL cholesterol. N. Engl. J. Med..

[26] Mitchell T (2014). Pharmacologic profile of the Adnectin BMS-962476, a small protein biologic alternative to PCSK9 antibodies for low-density lipoprotein lowering. J. Pharmacol. Exp. Ther..

[27] Zhang Y (2014). Identification of a small peptide that inhibits PCSK9 protein binding to the low density lipoprotein receptor. J. Biol. Chem..

[28] Frank-Kamenetsky M (2008). Therapeutic RNAi targeting PCSK9 acutely lowers plasma cholesterol in rodents and LDL cholesterol in nonhuman primates. Proc. Natl. Acad. Sci. USA.

[29] Fitzgerald K (2014). Effect of an RNA interference drug on the synthesis of proprotein convertase subtilisin/kexin type 9 (PCSK9) and the concentration of serum LDL cholesterol in healthy volunteers: A randomised, single-blind, placebo-controlled, phase 1 trial. Lancet.

[30] Marshall JC (2002). Measurement of endotoxin activity in critically ill patients using whole blood neutrophil dependent chemiluminescence. Crit. care.

[31] Charbonney E (2016). Endotoxemia Following Multiple Trauma: Risk Factors and Prognostic Implications. Crit. Care Med..

[32] Go LL, Healey PJ, Watkins SC, Simmons RL, Rowe MI (1995). The Effect of Endotoxin on Intestinal Mucosal Permeability to Bacteria *In Vitro*. Arch. Surg..

[33] Guo S, Al-Sadi R, Said HM, Ma TY (2013). Lipopolysaccharide Causes an Increase in Intestinal Tight Junction Permeability *in Vitro* and *in Vivo* by Inducing Enterocyte Membrane Expression and Localization of TLR-4 and CD14. Am. J. Pathol..

[34] Shenep JL, Flynn PM, Barrett FF, Stidham GL, Westenkirchner DF (1988). Serial Quantitation of Endotoxemia and Bacteremia During Therapy for Gram-Negative Bacterial Sepsis. J. Infect. Dis..

[35] Vianna RCS (2004). Antibiotic Treatment In A Murine Model Of Sepsis: Impact On Cytokines And Endotoxin Release. Shock.

[36] Dwivedi DJ (2016). Differential Expression of PCSK9 Modulates Infection, Inflammation, and Coagulation in a Murine Model of Sepsis. Shock.

[37] Pirro M (2017). Hepatitis C virus and proprotein convertase subtilisin/kexin type 9: a detrimental interaction to increase viral infectivity and disrupt lipid metabolism. J. Cell. Mol. Med..

[38] Ricci C (2018). PCSK9 induces a pro-inflammatory response in macrophages. Sci. Rep..

[39] Paciullo F (2017). PCSK9 at the crossroad of cholesterol metabolism and immune function during infections. J. Cell. Physiol..

[40] American College of Chest Physicians/Society of Critical Care Medicine Consensus Conference: Definitions for sepsis and organ failure and guidelines for the use of innovative therapies in sepsis. *Crit. Care Med*. **20**, 864–874 (1992). 1597042

