# Supplementary material for: Reduced Proprotein convertase subtilisin/kexin 9 (PCSK9) function increases lipoteichoic acid clearance and improves outcomes in Gram positive septic shock patients
Source: Sci Rep. 2019 Jul 22;9:10588. doi: 10.1038/s41598-019-46745-0 (PMC6646337; doi:10.1038/s41598-019-46745-0)
Supplement: Supplementary file 1 — Supplemental Information [file 41598_2019_46745_MOESM1_ESM.docx]

**Supplemental Information**

**Reduced Proprotein convertase subtilisin/kexin 9 (PCSK9) function increases lipoteichoic acid clearance and improves outcomes in Gram positive septic shock patients**

Alex K. K. Leung, Kelly Roveran Genga, Elena Topchiy, Mihai Cirstea, Tadanaga Shimada, Chris Fjell, James A. Russell, John H. Boyd, Keith R. Walley*

Centre for Heart Lung Innovation, St. Paul's Hospital, The University of British Columbia, Vancouver, BC, Canada.

Correspondence to: Keith R. Walley

HLI, St. Paul's Hospital, 1081 Burrard Street. Vancouver, BC, Canada V6Z 1Y6

Phone: (604) 806-8136, Fax: (604) 806-8351

Email: [Keith.Walley@hli.ubc.ca](mailto:Keith.Walley@hli.ubc.ca)

**MATERIALS AND METHODS**

*Cells and Materials.* HepG2 hepatocytes (American Type Culture Collection) were cultured in DMEM medium supplemented with 10% fetal bovine serum. HEK 293 cells co-transfected with human TLR2 and secreted alkaline phosphatase (SEAP) reporter gene coupled to the NF-κB/AP-1 pathway (HEK-Blue-hTLR2) were purchased from InVivoGen (San Diego, CA) and were maintained according to the manufacturer's protocol. All cells were incubated at 37^o^C with 5% CO_2_. All cell culture reagents were obtained from Invitrogen (Carlsbad, CA). Lipoteichoic acid was obtained from Sigma-Aldrich (St. Louis, MO) and was used without any further purification. The fluorescent label BODIPY-FL 4-sulfotetrafluorophenyl ester (BODIPY-FL-STP) was purchased from ThermoFisher Scientific (Waltham, MA). Sephadex G-50 gel filtration beads and the pyrogen-free water used throughout the experiment were obtained from Sigma-Aldrich (St. Louis, MO). Purified recombinant human PCSK9 were synthesized by Thermos Scientific (Waltham, MA) using the mRNA sequence of human PCSK9 available from the National Center for Biotechnology Inoformation (NCBI, Bethesda, MD, Accession number NM_174936.3). The NCBI sequence contains two mutations, E670G and I474V, and were replaced with the correct amino acid residue in the wildtype PCSK9 protein used in this study. The 3 PCSK9 loss-of-function variants (rs11591147 R46L, rs11583680 A53V, rs562556 V474I) were synthesized from the wildtype sequence with the corresponding amino acid substitution. All recombinant PCSK9 proteins were synthesized with N-terminal His-tags that have been removed post-translationally by Tobacco Etch Virus (TEV) protease.

*Fluorescent Labeling of LTA.* Lipoteichoic acid was conjugated to the fluorescent label BODIPY-FL-STP using a modified version of the methods described by Levels et al.^9^ Briefly, LTA was first dissolved in pyrogen-free water at a concentration of 2 mg/mL, while a stock solution of BODIPY-FL-STP in pyrogen-free water was prepared at a concentration of 10 mg/mL. A molecular mass of 8 kDa was used as the average molecular weight of the LTA monomer.^9^ LTA at a concentration of 0.5 mg/mL was conjugated with 0.338 mg/mL of BODIPY-FL-STP in 100 mM sodium bicarbonate buffer at pH 8.3 at room temperature for 2 hours in the dark. Glycine was added to the reaction mixture at a final concentration of the 25 mM and incubated for an additional 30 min to remove any non-conjugated BODIPY-FL-STP. The BODIPY-LTA was separated from BODIPY-glycine by gel filtration on a 7 mL Sephadex G-15 column. The concentration of LTA in each fraction was determine using the HEK Blue hTLR2 assay described below and the fluorescent intensity of each fraction was determined using the Spectramax i3 fluorescent plate reader at λ_ex/em_ = 500/525 nm.

*HEK Blue hTLR2 Assay.* HEK 293 cells co-transfected with human TLR2 (hTLR2) and a SEAP reporter gene coupled to the NF-κB/AP-1 pathway was used to determine the active concentration of LTA after conjugation with BODIPY-FL-STP. The amount of SEAP secreted is proportional to the degree of hTLR2 activation and can be detected colorimetrically with HEK-Blue Detection cell culture medium (Invivogen, San Diego, CA). HEK-Blue hTLR2 cells were plated onto a 96-well plate at 50000 cells/well along with 20 μL of LTA fractions in each well. A standard curve was prepared using non-conjugated LTA to determine the LTA concentration in each fraction. The plate was incubated at 37^o^C, 5% CO_2_ and 100% humidity for 18 h. Absorbance readings were measured at 650 nm using a plate reader.

*Effect of PCSK9 on the uptake of LTA by hepatocytes by flow cytometry.* Immortalized human hepatocytes (HepG2 cell line, ATCC) were seeded into a 24-well plate and grown to 80% confluence. The culture medium for the duration of the experiment was 80% DMEM (Invitrogen 11965-065) and 20% human plasma from pooled healthy donors. Cells were pre-treated with 0, 100, 300, 1,000, 3,000 and 10,000 ng/mL of either recombinant wild-type human PCSK9, or various loss-of-function PCSK9 variants for 2 h prior to LTA treatment

Cells were treated with BODIPY-FL-LTA at 1 µg/mL. After 24 h of LPS treatment, each well was rinsed twice with PBS and detached with Accutase (BD Biosciences, San Jose, CA). The cells were collected, washed with PBS, then resuspended in 500 μl of PBS and analysed via flow cytometry (GalliosTM Flow Cytometer, Beckman Coulter, Brea, CA). Cells were gated via forward and side scatter for viability using previously determined parameters; 5000 gated cells were counted per sample. The output of interest was the median fluorescence intensity from the instrument’s FL1 laser (ex: 488 nm, em: 525/20 nm). Background autofluorescence of cells not treated with BODIPY-FL-LTA was subtracted to determine the fluorescence level resulting from the uptake of the LTA conjugate. Data analysis was performed using Kaluza Analysis 1.3 software (Beckman Coulter, Brea, CA).

*Confocal Microscopy.* Cells were seeded onto coverslips at 50,000 cells/well in 24-well cell culture plates and grown for 2 days. Cells were first pre-treated with 10 μg/mL of either wild-type or various loss-of-function variants of human PCSK9 for 4 h. Cells were then treated with 2 µg/mL of BODIPY-FL-LTA for 4 h. The culture medium for the duration of the experiment was DMEM (Invitrogen 11965-065) supplemented with 10% fetal bovine serum. Cells were washed once with PBSCM (1x PBS supplemented with 1 mM MgCl_2_ and 0.1 mM CaCl_2_) and fixed with 4% paraformaldehyde containing 1 μg/mL Hoescht 33342 (ThermoFisher Scientific, Waltham, MA) for 30 minutes. Coverslips were washed extensively with PBSCM and water to reduce background. Coverslips were mounted onto glass slides and examined with confocal microscopy (Zeiss LSM 880). Fluorochromes were excited at 405 nm (Hoescht 33342) and 488 nm (BODIPY-FL) and images were collected with a 62x oil-immersion objective lens.

*Vasopressin and Septic Shock Trial (VASST) Derivation Cohort.* VASST was a multicenter, randomized, double blind, controlled trial evaluating the efficacy of vasopressin versus norepinephrine in 778 patients who had septic shock,^40^ and vasopressor infusion of at least 5 μg/min of norepinephrine, or equivalent.^12^ Inclusion criteria and clinical phenotyping are described elsewhere.^12^ DNA was available from 632 patients. The research ethics boards of all participating institutions approved this trial and written informed consent was obtained from all patients or their authorized representatives. The research ethics board at the coordinating centre (University of British Columbia) approved the genetic analysis.

*St. Paul’s Hospital (SPH) Validation Cohort.* All patients admitted to the ICU at SPH in Vancouver, Canada between July 2000 and January 2004 were screened and of these, 415 patients were classified as having septic shock, had DNA available, and were successfully genotyped. Septic shock was defined by the presence of two or more diagnostic criteria for the systemic inflammatory response syndrome, proven or suspected infection, new dysfunction of at least one organ, and hypotension despite adequate fluid resuscitation.^40^ Inclusion criteria and clinical phenotyping are described elsewhere.^41^ The Institutional Review Board at SPH and the University of British Columbia approved the study.

*Human Genotyping and SNP Selection.* DNA was extracted from buffy coat of discarded blood samples using a QIAamp DNA Blood Midi Kit (Qiagen) (VASST cohort) or a QIAamp DNA maxi kit (Qiagen, Mississauga, Canada) (SPH cohort). Known PCSK9 loss-of-function SNPs (rs11591147 R46L, rs11583680 A53V, rs562556 V474I), a known PCSK9 gain-of-function SNP (rs505151 G670E) and LDL receptor rs688 were genotyped in the VASST cohort as part of whole genome genotyping using the Illumina Human 1M-Duo genotyping platform (Illumina Inc.). rs11591147, rs11583680, rs562556, and rs505151 were genotyped in the SPH septic shock cohort using a Sequenom iPLEX Gold Assay (Genome Quebec, PQ, Canada). Patients were categorized as LOF carriers if they carried one or more minor allele of the 3 LOF SNPs (rs562556, rs11583680, and rs11591147) and no copies of the minor allele for the GOF SNP rs505151.

The human PCSK9 gene has been highly characterized and several relatively common missense variants (Minor Allele Frequency [MAF] ≥0.5%) and many rare missense and nonsense variants have been identified that are associated with decreased LDL levels, as an indicator of Loss-Of-Function (LOF) of PCSK9. Accordingly, we genotyped the relatively common PCSK9 LOF variants (MAP≥0.5%, rs11591147 R46L, rs11583680 A53V, rs562556 V474I) in the VASST cohort. We compared this to a PCSK9 tag SNP, rs644000. All SNPs tested were in Hardy-Weinberg equilibrium.

We found that the LOF alleles of rs11591147, rs11583680, and rs562556 all preferentially segregated with the minor G allele of our tag SNP, rs644000 (**Table S1**). Out of 1324 total observed haplotypes a LOF allele was observed 442 times within 309 haplotypes. 83.5% of these LOF alleles were contained within haplotypes that also contained the rs644000 minor G allele. Only 15.5% of the LOF alleles were contained within rs644000 major A allele haplotypes. Thus, the minor allele of rs644000 is a marker of the most common PCSK9 LOF genetic variants.

| Haplotype | rs11591147 LOF R46L | rs11583680 LOF A53V | rs644000 Best tag SNP | rs562556 LOF V474I | rs505151 GOF G670E | Number of haplotypes in VASST | Percent haplotypes in VASST |
| --- | --- | --- | --- | --- | --- | --- | --- |
| 1 | C | G | A | A | A | 735 | 55.5% |
| 2 | C | G | A | A | G | 59 | 4.5% |
| 3 | C | A | A | A | A | 6 | 0.5% |
| 4 | C | G | A | G | A | 57 | 4.3% |
| 5 | C | G | G | A | A | 151 | 11.4% |
| 6 | C | G | G | G | A | 108 | 8.2% |
| 7 | C | A | G | A | A | 136 | 10.3% |
| 8 | C | A | G | G | A | 54 | 4.1% |
| 9 | A | G | G | G | A | 6 | 0.5% |
|  |  |  |  |  | rare haplotypes | | 0.9% |

**Table S1.** Relationship between PCSK9 Loss-Of-Function (LOF) variants (MAF≥0.5%) and rs644000G/A minor allele G. Haplotypes were resolved in VASST using PHASE* and haplotypes with MAP≥0.5% are displayed. Minor allele is colored for each SNP; LOF yellow, GOF green, rs644000 orange.

*. Stephens M, Donnelly P. A comparison of bayesian methods for haplotype reconstruction from population genotype data. *Am J Hum Genet.* 2003; 73: 1162-1169.

*Statistical Analysis.* We tested SNPs for Hardy-Weinberg equilibrium using a χ^2^ test. For human septic shock our primary analysis of 28-day survival curves used a log rank test stratified by Caucasian or non-Caucasian ancestry and a secondary analysis used logistic regression to examine 28-day survival by *PCSK9* genotype, including the covariates of age, gender, Caucasian ancestry, and a surgical versus medical primary diagnosis in the statistical model.

Patients were categorized as LOF carriers if they carried one or more minor allele of the 3 LOF SNPs (rs562556, rs11583680, and rs11591147) and no copies of the minor allele for the GOF SNP rs505151. All analyses were performed using R (version 2.8.1, www.R-project.org), SPSS version 22.0 (SPSS Inc, Chicago, IL), or Stata 12.0 (College Station, TX) statistical software packages.

**SUPPLEMENTAL FIGURE 1**

**Supplmental Figure 1** Survival over 28 days is illustrated in patients who were homozygous for the minor allele of rs688; rendering LDL receptor insensitive to PCSK9. Patients who carried at least one LOF allele of PCSK9 (dashed line) had no difference in 28-day survival compared to patients with the wildtype PCSK9 genotype (solid line).

**SUPPLEMENTAL FIGURE 2**

**
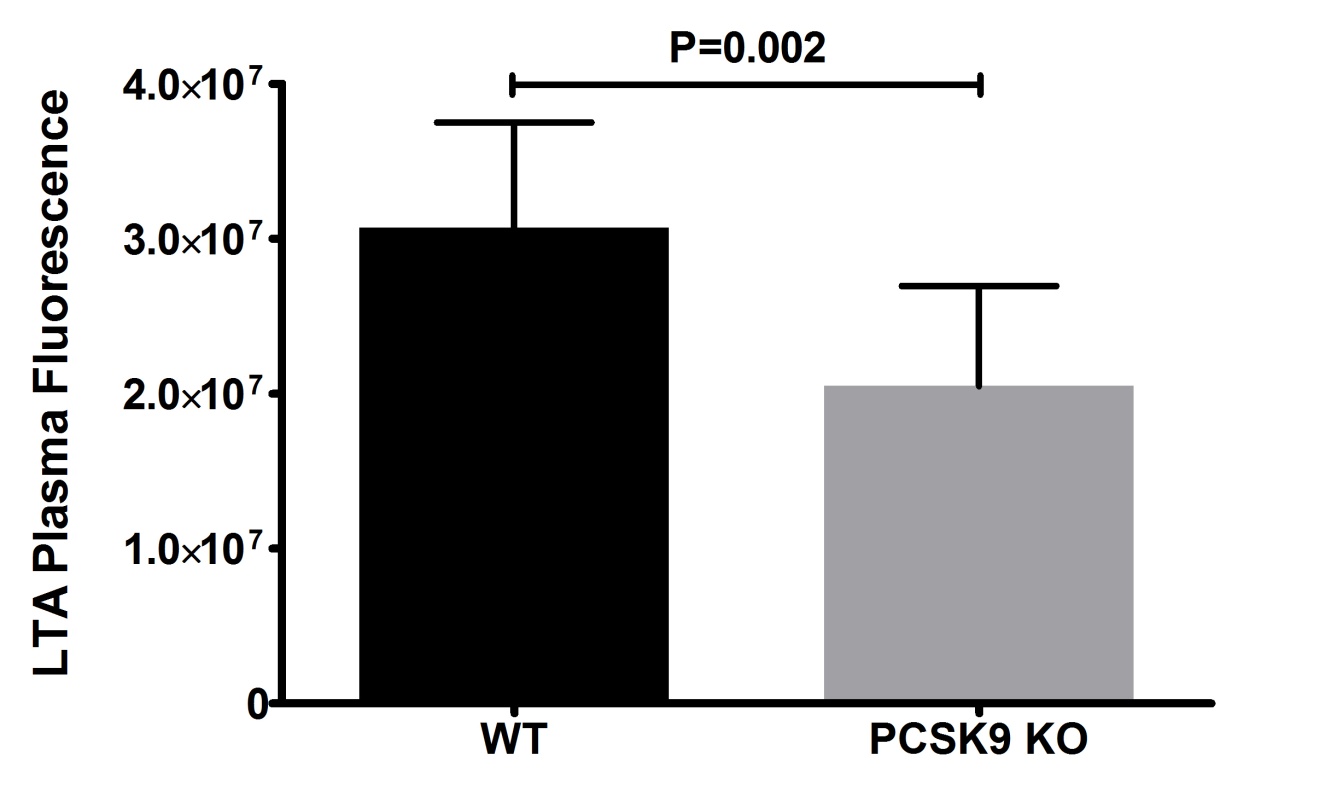
**

**Supplemental Figure 2** *PCSK9-/-* mice showed improved plasma clearance of intravenously administered LTA**.** PCSK-/- mice and wildtype litter-mate controls were injected with 0.3 mg/kg of BODIPY-FL-LTA intravenously via the tail vein. Blood was collected by cardiac puncture 6 hours following LTA administration and plasma fluorescence were measured at at λ_ex/em_ = 500/525 nm.
